# Genetics and Gene-Environment Interactions in Childhood and Adult Onset Asthma

**DOI:** 10.3389/fped.2019.00499

**Published:** 2019-12-11

**Authors:** Eva Morales, David Duffy

**Affiliations:** ^1^Biomedical Research Institute of Murcia (IMIB-Arrixaca), University of Murcia, Murcia, Spain; ^2^CIBER Epidemiología y Salud Pública (CIBERESP), Madrid, Spain; ^3^QIMR Berghofer Medical Research Institute, Brisbane, QLD, Australia

**Keywords:** asthma, genetics, environmental exposures, gene environment interactions, childhood, adulthood

## Abstract

Asthma is a heterogeneous disease that results from the complex interaction between genetic factors and environmental exposures that occur at critical periods throughout life. It seems plausible to regard childhood-onset and adult-onset asthma as different entities, each with a different pathophysiology, trajectory, and outcome. This review provides an overview about the role of genetics and gene-environment interactions in these two conditions. Looking at the genetic overlap between childhood and adult onset disease gives one window into whether there is a correlation, as well as to mechanism. A second window is offered by the genetics of the relationship between each type of asthma and other phenotypes e.g., obesity, chronic obstructive pulmonary disease (COPD), atopy, vitamin D levels, and inflammatory and immune status; and third, the genetic-specific responses to the many environmental exposures that influence risk throughout life, and particularly those that occur during early-life development. These represent a large number of possible combinations of genetic and environmental factors, at least 150 known genetic loci *vs*. tobacco smoke, outdoor air pollutants, indoor exposures, farming environment, and microbial exposures. Considering time of asthma onset extends the two-dimensional problem of gene-environment interactions to a three-dimensional problem, since identified gene-environment interactions seldom replicate for childhood and adult asthma, which suggests that asthma susceptibility to environmental exposures may biologically differ from early life to adulthood as a result of different pathways and mechanisms of the disease.

## Introduction

Asthma is characterized by a significant heterogeneity in relation to age of onset, clinical manifestations, genetics, environmental risk factors, response to treatments, and prognosis. Asthma affects as many as 339 million people worldwide (1), of whom 33% are under the age of 14, 27% are adults who first experienced symptoms in childhood (2), and 40% adult-onset cases. What proportion of this last group has the same underlying disease processes acting as in the first two groups? Is the “Dutch” hypothesis of a continuum from asthma to chronic obstructive pulmonary disease (COPD) a better description? (3). Similarly, there is much current interest in the relationship between childhood asthma and obesity. It has been suggested that obesity might affect childhood and adult-onset asthma by separate pathways (4), but evidence for this is still patchy. Allergy plays a key role in childhood-onset asthma, and these subjects more frequently have atopic dermatitis, hay fever and a family history of atopy in comparison to those who develop asthma in adulthood (5). Adult-onset asthma is characterized by reduced lung function and poor prognosis (6). So what proportion of adult-onset asthma has an atopic contribution? Our current understanding is that we need to comprehend both genetic factors, environmental exposures and their interactions.

Asthma runs strongly in families and estimates of its heritability range from 35 to 70%, showing higher estimates among boys and early-onset cases (7, 8). The study of asthma genetics has offered the possibility of understanding the causes and biological mechanisms of the disease as well as the identification of potential targets for treatment, although identified multiple loci only explain a limited proportion of asthma heritability (9).

Differences in rates of asthma between countries and its increasing prevalence in the past few decades clearly suggest that environmental exposures have an important role on asthma occurrence. Environmental risk factors of asthma include exposure to tobacco smoke, farm animals and related products, domestics cats, respiratory viral infections, microbial exposures, dietary factors, breastfeeding, medication, occupational exposures, indoor and outdoor air pollution, and diverse allergens (10). Environmental exposures, including those beginning in early life, play a pivotal role and the exact timing of exposure at critical windows of development influences genetic specific responses and individual risk trajectories that ultimately lead to the development of asthma (11, 12). Gene-environment interaction studies aim to explain how the strength and direction of associations between certain genetic variants and asthma may depend on given environmental exposures, and *vice versa*, and might explain in part the hidden heritability of asthma.

In the light of clinical and epidemiological importance of asthma and the potential benefits of further research into its etiology, this review will provide an overview on current understanding of genetics and gene-environment interactions in childhood and adult onset of the disease.

## Genetics of Childhood and Adult Onset Asthma

We first examine evidence from: (a) multivariate family based studies, where two traits e.g., childhood-onset asthma and adult-onset asthma are measured in the same family and the correlations between family members (who might be twins) across traits are interpreted; (b) the counting of overlapping genome-wide significant simple nucleotide polymorphism (SNPs) regression results from genome-wide association studies (GWAS) of each trait; (c) the accuracy of a genetic risk score generated from a GWAS of one trait in predicting the second trait in a second GWAS; (d) methods combining all SNPs from two GWAS to estimate the genetic correlation between traits; and (e) Mendelian Randomization (MR) studies of asthma risk factors, where genetic variants affecting the risk factor are tested for an association for asthma, thus increasing evidence of causation between the risk factor and asthma.

Childhood-onset asthma is most commonly accompanied by atopy. We know from a myriad of family-based studies, and now from large genotyping studies of unrelated individuals, that the diseases of the atopic triad are strongly heritable, and genetically correlated. Heritability is a measure of what proportion of trait differences between individuals in a given population are due to causative genetic differences between those individuals (13). Being a proportion, interpretation must be fastidious. For example, in developed countries asthma incidence has increased several-fold over the last 100 years, which must be due to changing environmental exposures. However, heritability estimates have remained roughly constant (14), which implies that average difference between individuals in environmental exposures has not greatly changed (else the genetic proportion of difference would be diluted), and that these exposures affect all genotypes equally at the first approximation. This puts an upper limit on the importance of gene-environment interactions at the population level, but the niceties would take too long to explain. A genetic correlation is also a proportion, comparing the genetic contribution (in the sense above) shared by two traits to those that are unshared. The correlation may well be via a mechanistically uninteresting causative chain e.g., the genetic correlation (r_g_ = +0.21, SE = 0.03) (15) between asthma and depression in the UK Biobank population might be mediated by atopy genes predisposing to chronic asthma, which is depressing for some individuals due to limiting effects on lifestyle. The latter might be suggested by the fact that the genetic correlations for Type 2 diabetes and depression, BMI and depression, myocardial infarction and depression, and schizophrenia and depression are all of a similar magnitude.

The concepts of heritability and genetic correlation come from the theory of inheritance for quantitative traits which are assumed to be (roughly) normally distributed. The extension to binary traits such as asthma requires modification of this model for use in a logistic or probit regression framework.

**Figure 1:**
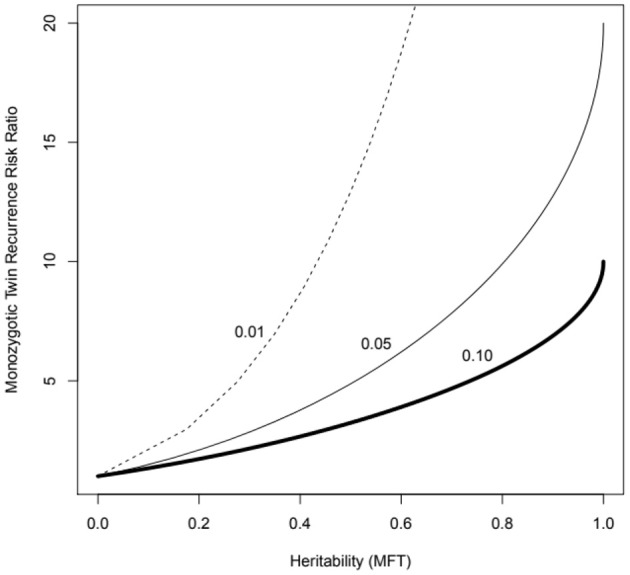
MZ twin recurrence risk ratio vs. heritability under the multifactorial threshold model (probit-normal mixed model, MFT) for three levels of population prevalence (1 to 10%), which might represent asthma of different levels of severity. Note that the recurrence risk ratio is bounded by the trait prevalence (e.g., cannot exceed 10 if the prevalence is 10%). The recurrence risk to an ordinary sibling will correspond to the value for half the heritability (the kinship coefficient for sibs is 0.5).

These methods deal easily with the usual case for complex diseases of very many contributing genes and can be calculated via trait correlations in pedigrees (including monozygotic—MZ, and dizygotic—DZ twins), as well as by measuring genotype-trait association directly (i.e., genome-wide association studies). They have a straightforward mathematical relationship to other measures such as the MZ twin recurrence risk ratio—the expected risk of disease of individuals genetically the same as asthma cases vs. whole population baseline risk (Figure 1).

Recently, we have seen the development of statistical methods to estimate heritability and genetic correlations in genome-wide association studies (GWAS) of multiple traits within the same study population, and even by combining separate univariate analyses of different populations using just summary data. These include the GCTA-COJO (16), LDAK Sumher (17), HESS (18) and LD score regression approaches (19). These approaches model contributions of loci of small effect that will not reach genome-wide statistical significance (*P* < 5 × 10^−8^) when tested one SNP at a time. Family-based methods will better estimate contributions of rare alleles, or alleles that are not well-measured by a particular set of genetic markers e.g., on a given SNP array, but sample sizes will usually be smaller when compared to genotyping studies of unrelated individuals.

In the case of Mendelian Randomization (MR) and related methods, the pattern of correlations between two traits and a measured SNP is interpreted knowing that genotype is both fixed at birth and largely unaffected by external confounders (ethnic confounding can be controlled for). MR analyses can involve one SNP at a time, a set of known associated SNPs from other studies, or whole genome; they can be bidirectional; and one of the traits can be gene transcript or expressed protein levels, simultaneously confirming the SNP to have biological effects on an intermediate trait and pointing to biological mechanisms for the trait-SNP correlation.

### Family Based Studies

A variety of recent studies generally confirm that the heritability of childhood-onset asthma is high, and that this tends to be higher than that of later-onset asthma (Table 1). The classical twin studies of children, where the family members being utilized are exactly the same age and birth cohort, tend to give the highest estimates. It should be noted that studies do not find an increased prevalence of asthma in twins, particularly MZ twins, especially after adjusting for covariates such as birth weight (20). Thomsen et al. (21) were able to break down that twin sample by age at onset of asthma, and found the heritability for onset after age 20 to be 60%, compared to 80% in younger twins. And data reported by Paaso et al. (5) can be reanalyzed to estimate the genetic correlations between parental allergy and adult-onset and childhood-onset offspring asthma (first two entries of Table 2). These give a consistent pattern, where the allergy early-asthma correlation is higher, but there is still a significant genetic correlation between atopy and later-onset asthma.

**Table 1:** Heritabilities of asthma from different studies using different designs and statistical methods.

**Trait**	**Heritability**	**References**	**Study type**
Asthma (<12 y.o.)	0.82 (0.79–0.85)	(20)	Twin study (12635 pairs)
Asthma (<20 y.o.)	0.78 (0.72–0.84)	(21)	Twin study (9051 pairs)
Asthma (>20 y.o.)	0.58 (0.58–0.82)	(8)	Twin study (11147 pairs)
Asthma (<13 y.o.)	0.46 (0.4–0.5)	(5)	Family study (1623 families?)
Asthma	0.42 (0.41–0.43)	(22)	Family study (128989 families, 481657 individuals).
Asthma	0.47 (0.23–0.72)	(23)	GWAS Bayesian mixture linear mixed model
Asthma (UKBB)	0.38 (0.35–0.41)	(24)	GWAS linear mixed model (GCTA)
Asthma (UKBB)	0.34 (0.32–0.36)	(25)	GWAS moment-matching LMM
Asthma (UKBB)	0.07 (0.05–0.08)	(26)	GWAS LD regression
Childhood-onset Asthma (UKBB)	0.004 (0.001–0.007)	Neale, 2018*^a^*	GWAS LD regression
Asthma onset (UKBB)	0.0 (−0.009 – 0)	Neale, 2018*^a^*	GWAS LD regression

a *http://www.nealelab.is/uk-biobank/*.

**Table 2:** Genetic correlations between asthma and related traits.

**Trait 1**	**Trait 2**	**Genetic correlation**	**References**	
Allergy (Espoo)	Childhood Asthma (Espoo)	~0.75	(5)	Family study
Allergy (Espoo)	Later Asthma (Espoo) >13 y.o.	~0.5	(5)	Family study
All Adult Asthma (UKBB)	Asthma (GABRIEL)	0.66	(26)	GWAS based LD regression
All Adult Asthma (UKBB)	Allergic Diseases (UKBB)	0.75	(26)	GWAS based LD regression
Asthma (GABRIEL)	Allergic Rhinitis	0.60	(27)	GWAS based LD regression
All Adult Asthma (UKBB)	Eosinophil count (UKBB)	0.45	(15)	GWAS based LD regression
All Adult Asthma (UKBB)	BMI (UKBB)	0.21	(15)	GWAS based LD regression
Childhood Asthma (GABRIEL)	BMI (GIANT Consortium)	0.19	(28)	GWAS based MoM method
All Adult Asthma (UKBB)	FEV_1_/FVC (UKBB)	−0.30	(15)	GWAS based LD regression
Adult Asthma	COPD	0.38	(29)	GWAS based LD regression

The heritability estimates based on GWAS (Table 1) tend to be lower, given they only represent the contributions of common measured SNPs (“array,” “chip,” or “SNP” heritability). Even so, the estimates from LD regression seem to be lower again.

### Overlap of Identified Causative Loci

The Trans-national Asthma Genetic Consortium (30) compared SNP allele frequencies in 24,000 asthma cases and 119,000 controls to detect 22 asthma associated loci. The locus of greatest effect was that on chromosome 17q21 in the region of *GSDMB* and *ORMDL3* genes. It has been known since 2008 (31) that this locus is associated with childhood onset asthma. Despite the association with early onset disease, these SNPs are not associated with atopy, and the asthma-increasing alleles probably reduce risk of other immunological conditions such as inflammatory bowel disease and Type 1 diabetes. If we take one of the functional 17q21 SNPs rs12936231 (actually within ZPBP2), in the UK BioBank the C allele increases “all” asthma OR = 1.1 (*P* = 1 × 10^−59^) and self-reported “emphysema/chronic bronchitis” OR = 1.07 (*P* = 1 × 10^−8^, but not doctor-diagnosed emphysema, *P* = 0.5), as well as neutrophil count (*P* = 7 × 10^−142^), but less strongly with eosinophil count (*P* = 3 × 10^−5^) (32). The effect sizes of these alleles are not large, so general inferences about childhood vs. adult asthma must be based on aggregated effects of multiple loci.

Demenais et al. (30) also confirmed the association of rs9272346 and rs9273349 in the region of HLA-DQ with asthma. These SNPs were reported more strongly correlated with adult-onset than childhood-onset disease (9, 33), but still exhibiting detectable association in early-onset cases. Like the 17q21 locus, are unrelated (or only weakly associated) to atopy. In the UKBB (32), the rs9272346^*^A allele increases incidence of asthma, coeliac disease, Type 1 diabetes, hypothyroidism, rheumatoid arthritis, and diminishes risk of multiple sclerosis and ulcerative colitis. It has no association with self-reported COPD. Pividori et al. (34), see below, classify it as shared by childhood and adult onset asthma in UKBB.

Ferreira et al. (35) carried out a GWAS meta-analysis (13 studies, total *N* = 360838) to detect 99 loci (136 peak SNPs) for atopic disease, defined as any of asthma, hayfever or atopic dermatitis. Of these, 49 were novel. Several loci were disease-specific, such as Filaggrin (FLG) for atopic dermatitis, or a SNP near IL18R1, where the effect allele was increased in asthma and hayfever, but not atopic dermatitis. Several variants were associated earlier onset of asthma (7445 asthma-only cases)—the strongest being rs921650 in GSDMB (17q21 region).

**Figure 2:**
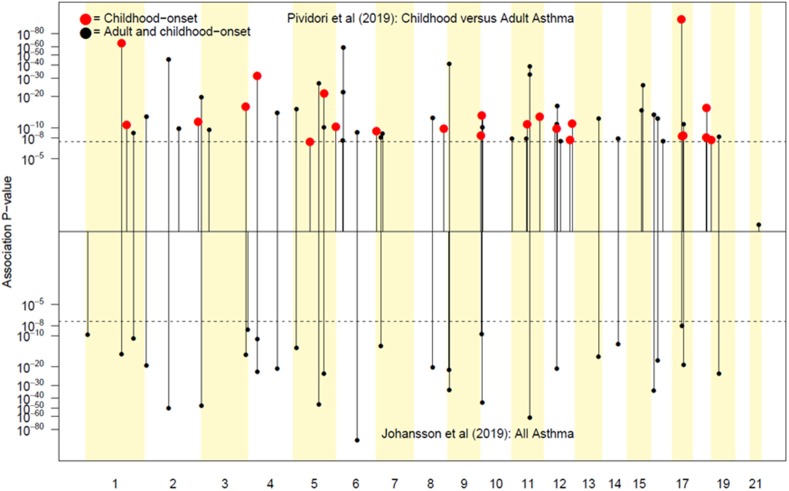
Top association peaks from Pividori et al. (34) and Johansson et al. (24).

**Figure 3:**
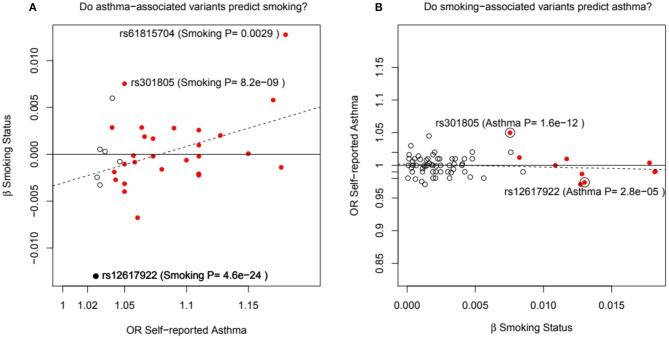
In **(A)**, the adult asthma SNP, rs12617922, from Pividori et al. (34), and all top asthma SNPs from Zhu et al. (26) vs. their relationship to smoking status in the UK Bio Bank analysis of Canela-Xandri et al. (32). Red color denotes an asthma association *P*-value in Canela-Xandri et al. (32) <5 × 10^−8^. For smoking status, only the *P*-values for rs12617922 and rs301805 are <5 × 10^−8^. The rs301805 SNP also shows a strong association with eosinophil count, and was flagged by Ferreira et al. (35) as an allergy locus. In **(B)**, smoking-associated SNPs [GWAS Catalog 2018] are tested—red color now denotes a smoking association *P*-value in Canela-Xandri et al. (32) < 5 × 10^−8^.

Pividori et al. (34) and Ferreira et al. (36) have recently reported on childhood and adult onset asthma within the UK Biobank (UKBB) sample. Pividori et al. were able to detect 61 independent asthma loci comparing 9433 childhood-onset cases, 21564 adult-onset cases, and 318237 controls. Of these, “23 were childhood onset specific, one was adult onset specific, and 37 were shared.” Ferreira and coworkers supplemented the UKBB data with data from 23andMe (further 32,000 childhood-onset cases and 215,000 controls) and reported 123 childhood-onset (age <20 years) asthma loci, of which 98 were reproducible in the second dataset. Five of these loci did not affect risk of adult-onset asthma. For adult-onset asthma, they found 34 replicable loci, of which 3 were significantly weaker as predictors of childhood disease. They estimated the genetic correlation between adult and childhood-onset asthma as 0.67. They further tested for loci specifically for age at diagnosis within their childhood-onset sample (defined as <20 years old) and concluded that such effects were relatively small (5% of variance, *P* = 0.02; but N was only 14,000, so power was low). There were 25 novel childhood-onset asthma loci detected, in or near biologically important genes such as *NOD2, IL4R, IL2RA*, and *IRF4*. Because the UKBB dataset is generally available, we can contrast these findings to those published by Zhu et al. (26) who reported a total of 38 loci for “doctor diagnosed” asthma detectable using an earlier available version of the dataset containing 7908 cases and 76768 controls, and Johansson et al. (24) who reported 52 loci defined via 41934 cases and 239773 controls (Figure 2). The “pure” adult-onset SNP reported by Pividori et al. was rs12617922 on chromosome 2 near *TEX41*, which decreased risk of tobacco smoking (*P* = 5 × 10^−24^), as commented on by Ferreira et al. (36). Figure 3 shows that such a strong negative association is not seen for any of the other asthma loci from Zhu et al. (26). A SNP in the same region near *TEX41*, rs10193706, was previously highlighted as associated with tobacco use in the subset of UKBB used for the BiLEVE study of COPD (37). In European populations, it is in moderate linkage disequilibrium with rs12617922 (*r*^2^ = 0.37), but not associated with self-reported asthma in the full UKBB sample.

An even more intriguing observation is that the peak SNP for this region in terms of “any” asthma and smoking (and suggestively with caffeine intake as well) is rs10427255, previously flagged as a locus for the photic sneeze reflex (*P* = 1 × 10^−11^) by Eriksson et al. (38) in the 23andMe dataset (see Table 3). These SNPs (rs12617922, rs10427255) are not associated with serum eosinophil level or atopy. Of the 32 SNPs known to be associated with photic sneeze reflex, only these near *TEX41* predict asthma, so there is no general relationship between these two phenotypes.

**Table 3:** Association between SNPs on chromosome 2q22.3 with self-reported asthma, tobacco use and coffee intake in the UK Biobank (32).

**SNP**	**Build 37 position**	**Allele**	**LD in Europeans (*****r***^****2****^**)**				**Smoking status**		**Current smoking**		**Coffee drinking**		**Self-reported asthma**	
							**Beta**	***P*-value**	**Beta**	***P***	**Beta**	***P***	**OR**	***P***
rs1533426	146119018	G	1				−0.013	1.0e-22	−0.0050	4.9e-06	−0.015	3.9e-05	1.03	8.4e-06
rs10427255	146125523	T	0.74	1			−0.013	9.5e-23	−0.0051	4.0e-06	−0.018	4.0e-05	1.03	6.9e-06
rs12617922	146156679	A	0.55	0.48	1		−0.013	4.6e-24	−0.0053	1.5e-06	−0.019	3.2e-06	1.03	2.8e-05
rs10193706	146316319	C	0.32	0.18	0.37	1	−0.013	8.9e-24	−0.0056	3.6e-07	−0.025	1.6e-09	1.02	0.01

*Both rs1533426 and rs10427255 (38) are associated with the photic sneeze reflex and rs10193706 was previously associated to heavy cigarette smoking (37)*.

Hobbs et al. (29) examined COPD in 25000 cases and 58000 controls. There were 22 associated loci (13 novel), none overlapping with asthma loci from the GABRIEL study or the NCBI GWAS catalog. Examining the UK BioBank via the GeneAtlas web site (32), one does find that five of their top COPD SNPs are associated with self-reported asthma at *P* < 1 × 10^−5^. The strongest association of these five is for rs2070600 (a protein coding change Gly82Ser in AGER on chromosome 6, *P* = 4 × 10^−29^), where the T (Serine) allele (European frequency 5–10%) increases asthma (OR = 1.15). The Hobbs et al. (29) result for COPD is OR = 1.21 (*P* = 6 × 10^−10^) for the C (Glycine) allele, so (if replicable) this would lead to a negative genetic correlation between asthma and COPD due to this locus. The Ser82 allele reduces sRAGE levels and increases FEV1 (39).

Hayden et al. (40) describe the COPDGene study of 10200 current and former adult smokers. The GWAS analysis found known childhood asthma risk SNPs were associated with asthma, and childhood asthma (7% of the sample) increased the risk of later COPD 3.4-fold, and also reduced lung function.

### Genetic Risk Scores

The components of the polygenic risk score (PRS) approach have already been presented (e.g., Figure 3), in that the PRS for a trait is merely the weighted sum of the regression coefficients for a number of significantly associated SNPs. Richardson et al. (41) have made available a web site presenting results of such cross-trait analyses (162 PRS, 551 outcomes) using the UK Biobank dataset (see Table 4). For example, while the BMI PRS predicts asthma, the asthma PRS does not predict BMI, a finding we can interpret as implying BMI is causative of asthma risk.

**Table 4:** Application of SNP-based Risk Scores derived for one trait to predict a second trait from http://mrcieu.mrsoftware.org/PRS_atlas/ (41).

**PRS trait**	**Second trait**	**N SNPs**	**Beta**	***P*-value**
Cigarettes/d	Asthma	44	0.004	0.43
Asthma	Cigarettes/d	42	−0.001	0.82
BMI	Asthma	251	0.025	4.1 × 10^−6^
Asthma	BMI	42	0.002	0.23
Asthma	“Chronic bronchitis/!!breakemphysema”	42	0.050	2.6 × 10^−4^
Major depression	Asthma	37	−0.005	0.37
Asthma	Depression	42	0.004	0.62

*The P-values test significance of the regression of the PRS on the second trait*.

A table of results for COPD PRS (spirometric phenotypes including FEV1/FVC were used to define COPD) has been presented by Shrine et al. (42) in yet another preprint. This score (294 SNPs) is a significantly predictor of self-reported asthma (*P* = 10^−41^). Examining the individual SNPs, we can see that 20 are significant (*P* < 1 × 10^−5^) independent predictors of UKBB self-reported asthma, and three are associated with hayfever/rhinitis (in *IL1RL1, SUOX* and *LRP1*).

### GWAS Genome-Wide Estimates of Genetic Correlations

The most widely used approach for this is the LD score regression method, and results from this are listed in Table 2. Speed and Balding (17) suggest that magnitude of estimates of genetic correlations tend to relatively similar across different methods, which is less true of heritability estimates, as seen above. We see that “any” asthma is more strongly correlated with atopic diseases such as allergic rhinitis (r_g_ 0.6–0.7) than it is with COPD (r_g_ 0.4).

### Mendelian Randomization Studies

Chen et al. (43) carried out a bidirectional MR analysis of the direction of causation between obesity and childhood asthma in Taiwan. While the obesity genetic risk score was a significant predictor of asthma, the asthma genetic risk score did not predict obesity. Granell et al. (44) similarly estimated the causal relative risk for the effect of BMI on asthma at 1.55 per kg/m^2^, slightly more strongly for non-atopic childhood asthma. In 162124 adults from European studies (45), the causal OR for BMI on asthma was 1.07 (−9 ml FEV1, 16 ml FVC). There was no causal effect on allergic sensitization or serum IgE level—despite a positive phenotypic correlation between IgE and overweight in their study population. After all the above, however, Contreras et al. (46) found in a traditional longitudinal study that asthma diagnosis by age 4 was associated with obesity at age 8, which would seem consistent with the idea that asthma causes obesity, or at the very least that genes causative of asthma also cause obesity via a different pathway (acting at a different age) than that by which they cause asthma. Does this give an insight into adult-onset asthma? Multiple longitudinal studies in adults have found that overweight precedes and predisposes to asthma. Burgess et al. (47) showed BMI at age 7 years predicted development of asthma with an onset after age 21 years. So, in this case a causal pathway from overweight to asthma would be supported by both genetic and non-genetic studies.

Finally, the relationship between vitamin D deficiency and childhood and adult asthma is still unclear, with both cross-sectional epidemiological and animal model evidence making this plausible but results from supplementation studies unsupportive (48). MR studies (49, 50) have showed no relationship between vitamin D level SNPs and asthma or atopy.

## Gene-Environment Interactions in Childhood and Adult Onset Asthma

The most frequently evaluated genetic-environment interactions on asthma include early-life and lifelong exposure to tobacco smoking, outdoor air pollutants, indoor exposures, a farming environment, and microbial exposures. The vast majority of the studies have applied a candidate gene approach and generally examined genes involved in antioxidant defenses, detoxification, inflammation, innate immunity, lung development, and epithelial function. Some investigations are based on family studies and few hypothesis-free studies, also called genome-wide interaction studies (GWISs), are available yet (Table 5).

**Table 5:** Summary of the most relevant examples of gene-by-environment interaction identified in asthma in epidemiological studies.

**Exposure**	**Study design**	**Gene/region (variant)**	**Gene/region functionality**	**Asthma onset**	**References**
**Tobacco smoking**					
Childhood ETS	Family study	Chr. 1p, 5q, and 17p	—	Childhood/adult	(51)
Childhood ETS	Family study	Chr. 5q	—	Childhood/adult	(52)
Childhood ETS	Family study	Chr. 1q43-q44, 4q34, 5p15, and 17p11	—	Childhood/adult	(53)
Childhood ETS	Family study	Chr. 17q21	Transcriptional activity of ZPBP2, GSDML and ORMDL3 genes	Childhood	(54)
Maternal smoking *in utero*	Candidate gene	*GSTM1 (deletion)*	Antioxidant defenses/detoxification	Childhood	(55) (56)
Childhood ETS	Candidate gene	*GSTM1 (deletion)*	Antioxidant defenses/detoxification	Childhood	(57)
Household ETS	Candidate gene	*GSTP1(Ile105Val)*	Antioxidant defenses/detoxification	Childhood	(58)
Maternal smoking during childhood	Candidate gene	*GSTP1 (Ile105Val)*	Antioxidant defenses/detoxification	Childhood	(59)
Maternal *in utero* smoking or first 2 months of age	Candidate gene	*GSTP1(Ile105Val)* *TNF-α(−857)*	Antioxidant defenses/detoxification Inflammation	Childhood	(60)
Parental smoking	Candidate gene	*TNF-α (−208, −308)*	Inflammation	Childhood	(61)
Maternal smoking *in utero*	Candidate gene	*IL1RN (rs2234678)*	Inflammation	Childhood	(62)
*In utero* ETS	Candidate gene	*TGFB1 (−509)*	Airway inflammation and remodeling	Childhood	(63)
Household ETS	Candidate gene	*IL-13*	Inflammation	Childhood	(64)
Childhood ETS	Candidate gene	*ICAM1 (rs5491, rs5498)*	Inflammation	Childhood	(65)
Ever smoking	Candidate gene	*IL-3 (rs40401)*	Leukotrienes, IL-4 and TNF-α release	Adult	(66)
*In utero* ETS	Candidate gene	*ADAM33*	Lung development Endothelial celldifferentiation	Childhood	(67)
Childhood ETS	Candidate gene	*CDH1 (−160)*	Epithelial function	Childhood	(68)
*In utero* ETS ChildhoodETS	GWIS	Chr. 18 near *EPB4IL3 (rs8094633)* *PACRG(rs1575472)*	Cell-cell junctions and lung development Motile cilia function andmorphogenesis	Childhood	(69)
Active tobacco smoking	GWIS	Intergenic regions on Chr. 9 and 12	Gene expression regulation in lungs	Adult	(70)
**Outdoor air pollutants**				
NOx and SO_2_	Candidate gene	*GSTP1 (Ile105Val)*	Antioxidant defenses/detoxification	Childhood	(71)
Ozone	Candidate gene	*GSTP1 (Ile105Val)* *GSTM1(deletion)*	Antioxidant defenses/detoxification	Childhood	(72)
Ozone and PM2.5	Candidate gene	*GSTP1 (Ile105Val)*	Antioxidant defenses/detoxification	Childhood	(73)
PM10	Candidate gene	*GSTP1 (Ile105Val)*	Antioxidant defenses/detoxification	Childhood	(74)
NO_2_	Candidate gene	*GSTP1 (Ile105Val, rs1138272)*	Antioxidant defenses/detoxification	Childhood	(75)
Major road length in 100-m buffer	Candidate gene	*GSTM1 (deletion)* *GSTT1(deletion)*	Antioxidant defenses/detoxification	Childhood	(76)
Living <200 m from a major road	Candidate gene	*GSTT1 (deletion)*	Antioxidant defenses/detoxification	Adult	(77)
NO2	Candidate gene	*NQO1 (rs2917666)*	Antioxidant defense	Adult	(78)
Ozone	Candidate gene	*TNF-α (−308)*	Inflammation	Childhood	(79)
PM2.5	Candidate gene	*TLR2 (rs4696480)* *TLR4 (rs2770150, rs10759931, rs6478317,rs1927911)*	Innate immunity	Childhood	(80)
NO_2_	GWIS	*BMGALT5* *ADCY2* *DLG2*	Glycosphingolipids synthesis Lung function Epithelialstructure	Childhood	(81)
**Indoor exposures**					
Mold/dampness	Candidate gene	*IL-4*	Humoral and adaptative immunity	Childhood	(82)
Gas cooking	Candidate gene	*GSTM1 (deletion)*	Antioxidant defenses/detoxification	Adult	(83)
Household carpet use	Candidate gene	*IL-13 (h1011 haplotype)*	Airway inflammation	Childhood	(84)
**Farming-related exposures**				
Farm milk consumption	Candidate gene	*CD14 (−1721)*	Innate immunity	Childhood	(85)
Living on farm	Candidate gene	*TLR2 (−16934)*	Innate immunity	Childhood	(86)
Farm environment	Candidate gene	*TLR6 (rs1039559, rs5743810)*	Innate immunity	Childhood	(87)
Farming exposures	GWIS	*GRM1*	Immunologic synapsis Th1 cytokineproduction	Childhood	(88)
**Microbial exposure**				
Early respiratory infections	Candidate gene	Chr. *17q21*	Transcriptional activity of ZPBP2, GSDML and ORMDL3	Childhood	(89)
Endotoxins	Candidate gene	*TLR4 (−299, −399)*	Innate immunity	Adult	(90)
Dust endotoxins	Candidate gene	*CD14 (−260)*	Innate immunity	Childhood/adult	(91)
Endotoxins	Candidate gene	*CD14 (−260)* *MD2(rs10808798)*	Innate immunity	Adult	(92)
Endotoxins	Candidate gene	*CD14 (−260)*	Innate immunity	Childhood	(93)

### Tobacco Smoking Exposure

This is the most well-studied environmental exposure with respect to genetic interactions in asthma in humans. Family studies have shown regions of linkage to asthma phenotypes to differ by environmental tobacco smoke (ETS) exposure in childhood (51–54). A study in 144 US families suggested that genes in chromosomal regions 1p, 5q, and 17p might interact with ETS to confer asthma risk in the exposed groups (51). The evidence for linkage for asthma and bronchial hyperresponsiveness (BHR) for chromosome 5q in the passive smoke-exposed groups during childhood was subsequently replicated in 200 Dutch families (52). A French study conducted in 295 families reported four regions, 1q43-q44, 4q34, 5p15, and 17p11, potentially involved in genetic susceptibility to BHR interacting with ETS in early life (53); however, none of these regions had been reported by previous genomes scans on gene-ETS interaction, except for the region in the 1q43-q44. Moreover, an interaction has been described between 17q21 variants and early-life exposure to ETS in early-onset asthma (54). The interaction between *ORMDL3* variants and early-life exposure to ETS in childhood-onset asthma was later replicated (94, 95).

Most candidate gene studies have focused on examining variants in genes coding for antioxidant defenses and xenobiotic-metabolizing enzymes and childhood-onset asthma susceptibility to tobacco smoking, especially the glutathione-S transferase (GST) family of antioxidant enzymes. Childhood-onset asthma risk has been shown to differ by *GSTM1* genotype in relation to both maternal smoking in pregnancy (55, 56) and childhood ETS exposure (57), with effects largely restricted to children with *GSTM1* null genotype. Results from other studies have suggested an interaction between *GSTP1* rs1695 A (Ile105) is a risk allele for childhood wheeze illness in relation to maternal smoking in early life, with an effect most clearly seen in children who are exposed to maternal smoking (59, 60). Most of the studies looking at interactions with *GSTM1* and *GSTP1* genetic variants have shown a positive finding, but not always in the same direction for *GSTP1* (58).

Given the importance of inflammation in asthma, genes related to inflammation responses and innate immunity have also been examined. Genetic variation in tumor necrosis factor (*TNF-*α) may contribute to childhood asthma and that associations may be modified by parental smoking (60, 61). Ramadas et al. (62) showed interaction of the interleukin-1 receptor antagonist (*IL1RN*) gene polymorphism rs2234678 and maternal smoking during pregnancy increased the risk for childhood asthma. Salam et al. (63) found that children with the transforming growth factor beta-1 (*TGFB1*)−509TT genotype are at increased risk of asthma when they are exposed to maternal smoking *in utero*; however, no interaction was found for parental tobacco smoke exposure in childhood (96). Household ETS and interleukin-13 gene (*IL-13*) variants may have interactive effects on childhood asthma phenotypes (64). Interactions between the human intercellular adhesion molecule 1 (*ICAM1*) polymorphisms and ETS have also been associated with risk of childhood-onset asthma (65). In adults, Miyake et al. revealed that the combination of ever smoking with interleukin 3 (*IL-3*) genetic variants was significantly positively associated with adult asthma in Japanese women (66).

Several studies have focused on genes related to epithelial function. Reijmerink et al. (67) first reported gene–environment interaction of *ADAM33* genotypes, the first identified asthma gene by positional cloning, and *in utero* tobacco smoke exposure with respect to childhood-onset asthma risk; however, no interaction was detected with postnatal ETS exposure (97). Moreover, Wang et al. (68) reported joint effects of ETS exposure and E-cadherin *CDH1* genotypes associated with the development of childhood asthma.

To date, two GWIS on tobacco smoke exposure and asthma are available. Scholtens et al. (69) conducted the first GWIS specifically aiming to identify genetic polymorphisms that interact with two well-known environmental risk factors for childhood-onset asthma: *in utero* and childhood tobacco smoke exposure. The authors found that genes reported previously to interact with tobacco smoke exposure with respect to asthma development (i.e., *GSTP1, TNF* and *ADAM33*) were not among the most significant hits and showed suggestive interactions between rs8094633 near erythrocyte membrane protein band 4.1 like 3 (*EPB41L3*) and exposure to *in utero* tobacco smoke, and between rs1575472 in parkin coregulated gene (*PACRG*) and childhood ETS. Interestingly, these two SNPS had not been identified previously in GWAS on childhood asthma. Subsequently, Vonk et al. conducted the first hypothesis-free genome-wide study to identify SNPs that interact with active tobacco smoking with respect to adult-onset asthma using data of the GABRIEL consortium, showing suggestive evidence for an interaction with two intergenic markers (rs9969775 on chromosome 9 and rs5011804 on chromosome 12) with potential regulatory functions linked to gene expression regulation in lung tissue (70).

Overall, existing literature has shown that prenatal and postnatal exposure to maternal/paternal smoking interacts with genetic variants to increase the risk of childhood-onset asthma; interestingly these genes are related to regions of linkage to asthma phenotypes and coding for antioxidant defenses and xenobiotic-metabolizing enzymes, inflammation responses and innate immunity and epithelial function. However, GWIS approaches have failed to replicate those findings revealing additional loci.

### Outdoor Air Pollutants

Interactions between outdoor air pollution and genetic variants have been focused on candidate genes related to antioxidative stress and detoxification systems, inflammation, and innate immunity as pathogenic pathways for asthma. Interactive effects of variants in genes belonging to the GST family (*GSTP1* Ile105Val*, GSTM1* deletion) with levels of different outdoor air pollutants (e.g., NO_2_, ozone, and particulate matter PM_10_ and PM_2.5_) have been described for childhood asthma (71–75) and traffic-related air pollution (76). Bowatte et al. (76) first reported significant effect modification of *GSTT1* polymorphisms for the association of traffic-related air pollution exposure and childhood-onset asthma, in contrast to previous studies that found no evidence of interaction (73, 74). In addition, Bowatte et al. also first demonstrated effect modification by *GSTT1* genetic variation, but not by *GSTM1* or *GSTP1* polymorphisms, on the association between traffic-related exposure and asthma in adults (77), in contrast to the study by Castro-Giner et al. that only detected an interaction with common polymorphisms in the *NQO1* gene (78).

Genetic variation in few genes related to inflammation and innate immunity have also been investigated as effect modifiers between outdoor air pollution and asthma. In the study of Li et al., the common TNF-α−308 GG genotype was found to interact with annual average levels of ozone in childhood onset asthma (79); although results were not replicated (75). Polymorphisms in toll-like receptor genes (*TLR2* and *TLR4*) have also been identified as potential effect modifiers of the association between outdoor PM2.5 levels and childhood asthma (80).

Recently, a GWIS of air pollution exposure and childhood asthma showed supportive evidence for interaction with outdoor NO_2_ levels for the novel loci *B4GALT5* and the previously lung disease associated loci *ADCY2* and *DLG2* (81).

In summary, results on interactions between exposure to outdoor air pollution and genetic variants on genes related to antioxidative stress, detoxification system, inflammation, and innate immunity on childhood and adult onset asthma are inconsistent across studies.

### Indoor Exposures

Several studies have investigated interaction between candidate genes and diverse indoor exposures. Gene-environment interactions between the IL-4 promoter and an indoor mold may play an important role in childhood asthma (82). Increased BHR in adults was associated with gas cooking (a major indoor source of NO2), but only among subjects with the *GSTM1* null genotype (83). IL-13 variants were found to interact with household carpet use on the risk of asthma in Taiwanese children (84).

### Farming-Related Exposures

The number of gene interactions with a farming environment remains very limited, focusing primarily genes related to innate immunity. Bieli et al. reported an interaction between early farm milk consumption and a polymorphism in the *CD14* gene (CD14/-1721) on the risk of childhood asthma (85). Effect modification by farm exposure in childhood on the association between polymorphism in *TLR* genes and asthma have been shown. Being exposed to a farm environment in childhood was protective against childhood-onset asthma for those with *TLR2*/-16934 T-allele (86), *TLR6*-rs1039559 T-allele and *TLR6*-rs5743810 C-allele (87). The most comprehensive GWIS in 1708 children from 4 rural regions of Central Europe for childhood asthma in relation to farm-related exposures did not reveal any significant interaction with common SNPs (88); however, strong interactions were found for rarer variants in 15 genes, particularly of the glutamate receptor, metabotropic 1 gene (*GRM1)*.

### Microbial Exposures

Respiratory viral infections in the first few years of life increase risk of childhood asthma; however, not all children develop the disease, suggesting an interaction with the host genetic factors (98). Smit et al. (89) found that 17q21 genetic variants enhance the association between early respiratory infections and childhood-onset asthma. The presence of two common polymorphisms in the extracellular domain of the *TLR4* has been found to modify the effect of endotoxins on asthma in adults (90). Moreover, multiple studies have characterized a similar interaction between asthma, *CD14* variants and environmental endotoxin exposure, a marker of microbial exposure capable of inducing severe airway inflammation. Zambelli-Weiner et al. found CD14/260 polymorphism to interact with dust endotoxin on the risk of childhood-onset asthma (91). Similarly, in adults, Smit et al. showed occupational endotoxin exposure and wheeze in agricultural workers to be significantly modified by genetic variants in *CD14* and *MD2* (92). A recent systematic-review has highlighted the apparent modification of the effect of early life endotoxin exposure on risk of asthma in childhood, but not in adults, by the CD14/260 polymorphism (93).

Literature on inhaled exposures such as indoor air pollution and farming-related is scarce and mostly focused on genetic interactions with genes related to innate immunity and childhood onset asthma. Host innate immunity genetic variation might play a key role in childhood and adult onset asthma susceptibility in relation to microbial exposures including endotoxins.

## Conclusions

**Figure 4:**
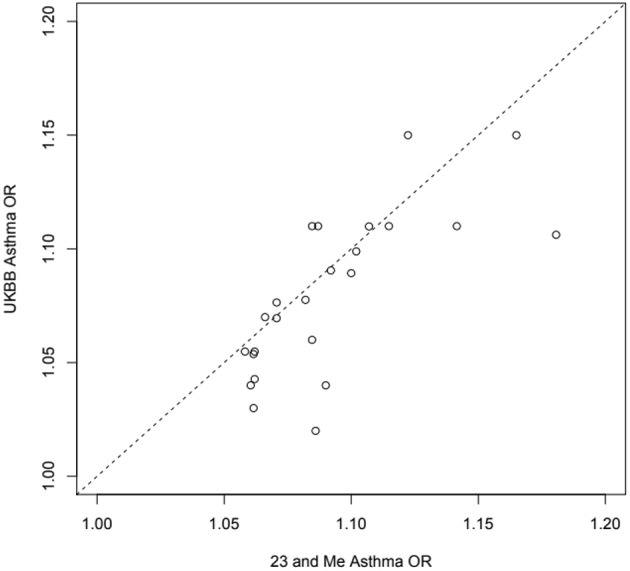
Estimated odds ratios for 24 independent top self-reported “any” asthma associated SNPs from 23 and Me (99) and the UK Biobank (32) samples: exact same SNP in or near *RORA, ZBTB10, ZPBP2, GATA3, ID2, IL33, CD247, TSLP, RAD50, HLAC, STAT6, D2HGDH, RAD51B, ADAMTS4, SMAD3, TLR1, BACH2, PEX14, ADORA1, TNFSF4, CDHR3, CLEC16A, LPP, LRRC32*.

There are relatively few large genetic studies examining adult-onset asthma. Studies often rely on relatively simple questionnaire based diagnostic criteria which are validated by the consistency of genetic associations detected (that is, diagnostic looseness is trumped by statistical power, see Figure 4), but might be less useful when trying to make fine distinctions about disease onset and persistence. In the present review we have attempted to triangulate via large studies of disorders that will overlap to greater and lesser extents with this condition. Specifically, this has been via the known fact that atopy is more strongly genetically correlated with childhood onset, and the likely fact that COPD overlap will be stronger for adult-onset asthma. In some cases, we can fix pathways of causation by using other types of study, such as traditional longitudinal studies, thus testing and constraining possible interpretations of the genetic evidence. Roughly, it seems that genetic causes of adult-onset asthma tend to affect childhood asthma to the same extent, which in hindsight seems very plausible. Childhood asthma has a larger contribution from atopy loci, while non-atopic immune-related genetic variants seem to be shared by adult-onset and childhood asthma. There are a few loci that might be specific to adult-onset disease, which may overlap with COPD e.g., AGER, but childhood asthma does seem to be a risk factor for adult COPD. Some constitutional risk factors such as increased BMI affect childhood-onset and adult-onset disease equally, and others such as vitamin D level do not show any genetic correlation with asthma.

Most of available studies on gene-environment interaction are focused on childhood-onset asthma, and few studies are based on adult subjects. Gene-environment interactions have revealed novel genes that have previously not been implicated in the pathogenesis of asthma; however, inconsistencies between studies and differences in direction of effects with specific genetic variants have been reported. These issues may likely be due to chance, insufficient power, different populations or ethnic origins, variation in study design and characterization of both exposures and disease phenotypes. Available GWISs have not replicated gene by environment interactions previously reported between common genetic variants and tobacco smoking, outdoor air pollutants, and farming-related exposures. Considering time of asthma onset extends the two-dimensional problem of gene-environment interactions to a three-dimensional problem, since identified gene-environment interactions seldom reproduce for childhood and adult asthma (e.g., endotoxin exposure and CD14/260 genotype). Therefore, evidence suggests that susceptibility of asthma to environmental exposures may biologically differ from early life to adulthood resulting in different pathways and mechanisms of the disease.

## Future Directions and Recommendations

- Genetic contributions are higher for childhood-onset asthma.
- The genetic overlap between childhood and adult onset asthma is large.
- The usually small size of the contribution of a single locus to heritability in the population does not preclude a large effect of a drug targeting that pathway. This is the justification given for these larger and larger studies that can detect smaller and smaller effects.
- Mendelian Randomization is a useful tool to investigate pathogenesis—if variation in a gene altering a putative intermediate variable is associated with risk, then environmental exposures affecting that same pathway are supported as truly causative.
- Lacking gene-environmental studies on important risk factors for asthma phenotypes such as diet, medication, microbiota, emerging pollutants, climate change and extreme weather conditions merit consideration.
- Despite methodological challenges, GWIS studies through collaboration hold promise for identifying unexpected gene environment interactions and improving our understanding of asthma phenotypes during a lifetime, beyond candidate studies based on our knowledge of biological processes and/or pathways.

## Author Contributions

All authors listed have made a substantial, direct and intellectual contribution to the work, and approved it for publication.

### Conflict of Interest

The authors declare that the research was conducted in the absence of any commercial or financial relationships that could be construed as a potential conflict of interest.
